# Association between Poor Outcomes and Risk of Refeeding Syndrome among Patients Urgently Admitted to the High Dependency Unit: A Single-Center Cohort Study in Japan

**DOI:** 10.3390/nu16193287

**Published:** 2024-09-28

**Authors:** Minoru Yoshida, Masako Suzuki, Haruaki Wakatake, Miyuki Kurisu, Hiroki Saito, Yuki Ohshima, Mayumi Kaneko, Kuniyasu Fujiwara, Yoshihiro Masui, Koichi Hayashi, Shigeki Fujitani

**Affiliations:** 1 Department of Emergency and Critical Care Medicine, St. Marianna University School of Medicine, 2-16-1 Sugao, Miyamae-ku, Kawasaki 216-8511, Kanagawa, Japan; minoru.yoshida@marianna-u.ac.jp (M.Y.); haru_waka_2110@marianna-u.ac.jp (H.W.); miyuki.kurisu@marianna-u.ac.jp (M.K.); hiroki.saito@marianna-u.ac.jp (H.S.); y2masui@marianna-u.ac.jp (Y.M.); kchhysh@gmail.com (K.H.); 2 Department of Nursing, St. Marianna University School of Medicine Yokohama Seibu Hospital, 1197-1 Yasashicho, Asahi-ku, Yokohama 241-0811, Kanagawa, Japan; masako.suzuki@marianna-u.ac.jp (M.S.); mayumi.kaneko@marianna-u.ac.jp (M.K.); kuniyasu.fujiwara@marianna-u.ac.jp (K.F.); 3 Department of Nutrition, St. Marianna University School of Medicine, 2-16-1 Sugao, Miyamae-ku, Kawasaki 216-8511, Kanagawa, Japan; yuki_ohshima.nu@marianna-u.ac.jp

**Keywords:** refeeding syndrome, nutrition assessment, high-dependency unit, electrolyte abnormality, in-hospital mortality, preventive strategy

## Abstract

**Background/Objectives:** Refeeding syndrome (RFS) is recognized as a potentially fatal metabolic disturbance, particularly concerning for non-critically ill patients who do not receive frequent electrolyte assessments. Assessing the risk of developing RFS and implementing preventive strategies is essential in these cases. We investigated the proportion of risk and its association with prognosis in a high-dependency unit (HDU). **Method:** This observational study was conducted in a tertiary care hospital’s HDU in Japan. We consecutively enrolled all patients who had been admitted urgently to the HDU and hospitalized for three days or more. We evaluated the National Institute for Health and Clinical Excellence (NICE) RFS risk factors at admission and classified patients into four groups based on the modified NICE criteria. The primary outcome was 30-day in-hospital mortality. The secondary outcome was a composite of 30-day in-hospital mortality and transfer to the intensive care unit, or discharge to locations other than home. Using logistic regression, we assessed the association between the four risk groups and outcomes, using the no-risk group as a reference. **Results:** A total of 955 patients were analyzed, of which 33.1%, 26.7%, 37.8%, and 2.4% were classified into the no-risk, low-risk, high-risk, and very high-risk groups, respectively. The 30-day in-hospital mortality was 4.4%, 5.5%, 5.0%, and 21.7%, respectively (Log-rank trend test: *p* = 0.047). In multivariable logistic regression, adjusting for sepsis, comorbidities, and age, only the very high-risk group was associated with 30-day in-hospital mortality (odds ratio: 5.54, 95% confidence interval: 1.73–17.79) A similar association was observed for the secondary outcomes. **Conclusions:** For patients admitted urgently to the HDU, there may be an opportunity to improve outcomes for very high-risk patients through preventive strategies.

## 1. Background

Refeeding syndrome (RFS) is characterized by metabolic disturbances involving electrolyte abnormalities and vitamin deficiencies [1]. It often develops after nutritional support is initiated in patients with malnutrition or who are in a hypercatabolic state [2,3]. One of the key pathophysiological aspects of RFS is that nutritional support triggers excessive insulin secretion, leading to low serum electrolyte levels due to a shift into the cells [1]. Additionally, phosphate is consumed during adenosine triphosphate production, making it more prone to depletion [4,5]. RFS including electrolyte abnormalities encompasses a wide range of critical symptoms, such as heart failure, respiratory failure, and muscle weakness [1,5,6]. Furthermore, some studies revealed that only refeeding electrolyte abnormalities without severe symptoms were associated with mortality [7,8,9]. In this way, electrolyte abnormalities such as hypophosphatemia play a crucial role in both risk assessment and diagnosis [5,6].

To prevent this potentially life-threatening condition, early assessment of risk factors and careful management of nutritional therapies are crucial [5,6]. The National Institute for Health and Clinical Excellence (NICE) has proposed RFS risk criteria, which include relatively easy-to-evaluate factors such as body mass index (BMI), weight loss, duration of little or no nutritional intake, and electrolyte abnormalities before reintroducing nutrition (Supplementary Table S1) [10]. These criteria were later revised by an expert consensus-supported algorithm, which classified the risks of RFS into four categories: no risk, low risk, high risk, and very high risk (modified NICE criteria) [6]. The incidence of RFS, however, varies depending on the definitions used and may differ not only among patient populations, such as those with different disease severities, but also among hospitals, races, and Japan’s aging society [2,11]. Accordingly, there is a pressing need for data that demonstrate the relationship between the incidence of RFS or its risk factors and patient outcomes, including mortality, within a consistent clinical setting.

There exists a heterogeneity in nutritional status among patients admitted to the emergency department. It is well established that RFS is more common and associated with higher mortality among intensive care unit (ICU) patients [2,8,9]. Recent application of the modified NICE criteria to our ICU patients revealed that approximately half fell into a high-risk or very high-risk group with a significant likelihood of developing RFS [12]. Moreover, 41.9% of these patients exhibited abnormal serum electrolyte levels—such as phosphate, potassium, or magnesium—which are critical for the development of RFS [5,6]. In contrast, the risk level for developing RFS among high-dependency unit (HDU) patients remains less clear. Blood sampling and electrolyte assessment are less frequent in HDUs and general wards compared to ICUs, making early detection of RFS less likely [13]. Thus, risk assessment at admission is crucial, yet there are few studies evaluating the incidence of RFS among urgently admitted patients at risk of developing RFS [14,15]. Furthermore, no studies have evaluated the impact of the modified NICE criteria on RFS development and outcomes in HDU admissions.

We therefore examined the prevalence of RFS risk categories based on modified NICE criteria and investigated which levels of NICE risk categories and risk factors were associated with mortality in patients requiring emergency HDU admission. The results were compared with those previously reported from ICU patients in our hospital [12]. This study aimed to identify patients at risk of developing RFS who require more focused attention and nutritional intervention among those urgently admitted to the HDU.

## 2. Materials and Methods

### 2.1. Study Design and Participants

Our retrospective observational study was conducted at the 24-bed HDU in St. Marianna University School of Medicine Yokohama Seibu Hospital, a tertiary care emergency center affiliated with the university hospital. This HDU admits patients who are less critically ill than those in the ICU but are not suitable for the general ward. Upon admission, we used a checklist to evaluate NICE RFS risk factors as part of nutritional screening and assessment for urgently admitted patients. The study received approval from the institutional review board at St. Marianna University School of Medicine (Kawasaki, Kanagawa, Japan, approval number: 5773) on 4 October 2022, and was conducted in accordance with the Declaration of Helsinki. Patient consent was waived due to the retrospective nature of the study, with opt-out information provided on our university website. Personal data were de-identified, and the study was registered with the University Hospital Medical Information Network (000054525).

**Figure 1:**
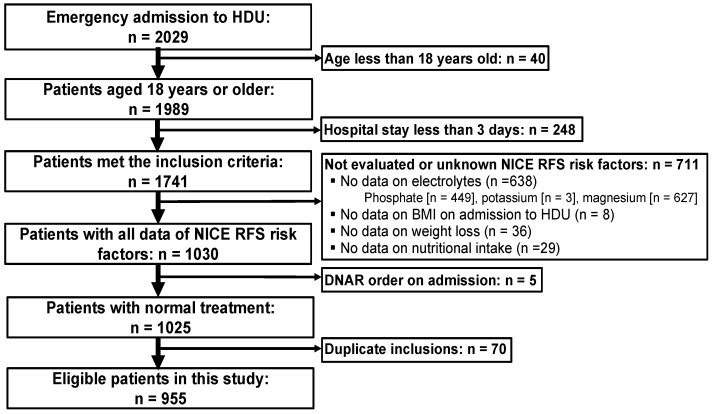
Patient enrollment criteria. BMI, body mass index; DNAR, do not attempt resuscitation; HDU, high dependency unit; NICE, National Institute for Health and Clinical Excellence; RFS, refeeding syndrome.

We consecutively enrolled patients aged 18 years or older who were admitted urgently to our hospital’s HDU between December 1, 2016 and April 30, 2019, and who stayed in hospital for at least 3 days (Figure 1). Patients with missing NICE RFS risk factors or who had expressed do-not-attempt-resuscitate orders were excluded. For patients admitted more than twice during the study period, only data from their first admission were included in the analysis.

### 2.2. Data Collection

To identify the patients at high risk of developing RFS upon admission to the HDU, registered nurses and dietitians assessed the NICE RFS risk factors (Supplementary Table S1) through interviews with patients or their family, using a checklist [10]. The nurses gathered information on changes in body weight (BW) over the 6 months prior to admission, nutritional intake before admission, and history of alcohol consumption from the patient, their family, or facility staff familiar with the patient’s background. On admission, BW and height were measured, and serum electrolytes (phosphate, potassium, and magnesium), insulin treatment, diuretic use, and chemotherapy were obtained from electronic medical records (EMRs). Alcohol abuse was defined as daily alcohol consumption of more than four standard drinks for men or more than three standard drinks for women [16]. Patients with various RFS risk factors were categorized into four groups based on NICE risk factors: no risk, low risk, high risk, and very high risk (modified NICE categories) [6]. Two nurses and one registered dietitian, certified as a specialist in nutritional support by the Japanese Society for Parenteral and Enteral Nutrition Therapy, reviewed and verified the medical records and laboratory data.

We also collected data on patients’ age, sex, Charleson Comorbidity Index (CCI) defined by ICD-10 codes [17,18], pre-existing illness (chronic kidney disease on dialysis, malignancy, psychiatric illness, and anorexia nervosa) that are considered to be associated with risk of developing RFS and electrolytes abnormality [5,11], admission type (medical or emergency surgery), location prior to admission, ambulance use, primary diagnosis on admission (cardiovascular, respiratory, digestive, neurology, infection, trauma, metabolic, hematologic, genitourinary, musculoskeletal and skin, or other medical conditions), and invasive treatments (such as mechanical ventilation with intubation and circulatory support). Only the primary diagnosis was recorded, and dual diagnoses were not included. In addition, we collected history of anorexia nervosa based on the *Diagnostic and Statistical Manual of Mental Disorders, fifth edition (DSM-5)* [19], as well as sepsis, defined as an infection with a Sequential Organ Failure Assessment score of 2 or higher on admission, according to Sepsis-3 [20], through a comprehensive EMR review. After admission to the HDU, the patients were managed with a variety of supportive care, and whether electrolyte supplementation was implemented was determined based on our protocol, which conformed to conventional clinical practices (Supplementary Table S2) [1,6,21,22].

### 2.3. Outcomes

The four RFS risk groups categorized by the modified NICE criteria served as the exposure variables. The primary outcome of this study was in-hospital mortality within 30 days of HDU admission. Secondary outcomes included the composite measure of in-hospital mortality, transfer to the ICU within 30 days of HDU admission, and discharge to locations other than home (e.g., transfer to another hospital or facility).

### 2.4. Sample Size

Based on results from our previous ICU study conducted at the same hospital [12], the multivariate analysis for 30-day mortality revealed the highest odds ratio in the very high-risk group compared to the no-risk group, although the difference was not statistically significant. To determine the required sample size, we used Lakatos’ method [23], estimating the survival rate of the no-risk group as 0.950 and the very high-risk group as 0.827, reflecting a 10% increase from the previous ICU study. The ratio of the very high-risk and no-risk groups was set to 0.08 (11 in the very high-risk group/140 in the no-risk group in the previous study) (Supplementary Table S3). Consequently, we calculated that 270 cases were needed for each group. Given that the ratio of the very high-risk group (*n* = 11) and the no-risk group (*n* = 140) to the total number of patients (*n* = 542) was 0.28 ([11 + 140]/542), the total number of cases required was calculated to be 964 (=270/0.28).

### 2.5. Statistical Analysis

Categorical data were presented as numbers and percentages and analyzed using the chi-square test. Continuous variables were expressed as medians with interquartile ranges (IQRs) and evaluated using the Kruskal–Wallis test. We assessed the proportion of identified NICE RFS risk factors and modified NICE categories using Pearson’s chi-square test to determine which risk factors were likely to contribute to the classification of risk groups.

Kaplan–Meier survival analysis was used to estimate survival probabilities for RFS risk groups, with in-hospital mortality censored 30 days after HDU admission. Survival curves for the risk groups were evaluated using the log-rank trend test. Univariate and multivariate analyses were conducted using logistic regression to compare the no-risk group as a reference with the other groups for primary and secondary outcomes, with results expressed as odds ratios (ORs) and 95% confidence intervals (CIs). We included the diagnosis of sepsis, CCI, and age as covariates due to their clinical importance. Patients with sepsis and a high burden of comorbidities generally had higher mortality rates [18,24,25] and were at an increased risk of developing RFS [21,26]. Advanced age was also considered a risk factor for RFS [11,27,28]. Not only from a clinical perspective but also by applying forward–backward stepwise selection, we ensured that the Akaike Information Criterion (AIC) was minimized [29,30]. The model with the smallest AIC was regarded as the primary analysis. To identify factors with significant association with primary and secondary outcomes, univariable logistic regression was performed. In addition, logistic regression analysis for the primary outcome was conducted by including age and sepsis alongside statistically significant risk factors from the univariate analysis. All analyses were conducted using JMP Pro (version 16. 2.0 for Windows, SAS Institute Inc., Cary, NC, USA), with statistical significance set at *p* < 0.05.

## 3. Results

### 3.1. Patient Characteristics

Among 2029 patients admitted urgently to our HDU from 1 December 2016 to 30 April 2019, we excluded 1074 based on the criteria mentioned (Figure 1). Of these, 248 patients were excluded due to a short hospital stay (i.e., less than three days), and 711 patients were excluded because at least one or more of the RFS risk factors were not evaluated. Ultimately, 955 cases were included for analysis.

Table 1 summarizes the characteristics of patients across various RFS risk categories. There was a significant difference in age among the four groups, with older ages in the low-risk to very high-risk groups compared to the no-risk group (*p* < 0.001). Body mass index (BMI) decreased as the risk increased (*p* < 0.001), with markedly smaller BMI observed in the very high-risk group. The median CCI was 1 across all groups, but in the no-risk group, the 25th percentile was 0, while it was 1 in other groups (*p* < 0.001). No patients in this study population were diagnosed with anorexia nervosa. The number of patients with sepsis tended to increase with higher risk levels (*p* = 0.059).

### 3.2. RFS Risk Categorization and Outcomes

**Figure 2:**
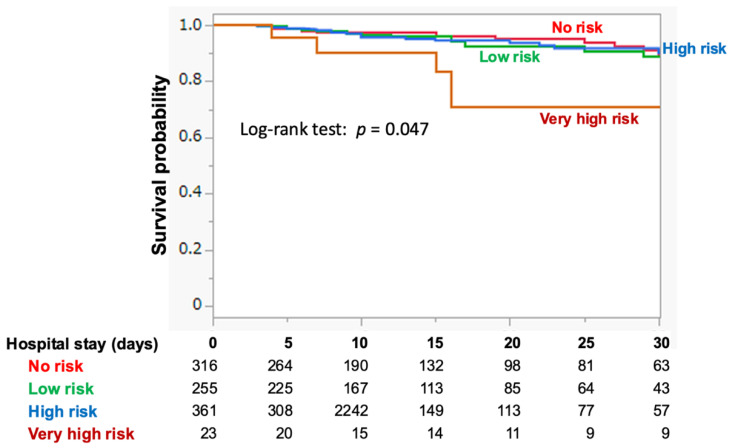
Kaplan-Meier survival analyses for mortality, with censoring at 30 days after HDU admission. The analyses show the survival probability for each risk group based on the modified NICE criteria. HDU, high dependency unit; NICE, National Institute for Health and Clinical Excellence.

The distribution of patients across the RFS risk categories of the no-, low-, and high-risk groups was comparable, but markedly lower in the very high-risk group (Table 2). There was a wide distribution of risk factors (e.g., BMI, BW loss, and reduced nutritional intake) among the risk categories. Patients with low electrolyte levels upon admission were predominantly found in the high-risk group. The very high RFS risk group exhibited the highest 30-day mortality rate (21.7%), whereas no significant difference in 30-day mortality was observed in the very high RFS risk group; no significant difference in 30-day mortality was observed between the high-risk group and the no-risk group (i.e., 5.0% vs. 4.4%). Kaplan–Meier analysis, with mortality censored at 30 days after HDU admission, showed a markedly lower survival probability over time in the very high-risk group, with no significant difference among the remaining groups (log-rank trend test: *p* = 0.047) (Figure 2). In multivariable logistic regression (primary analysis with the smallest AIC), adjusting for sepsis, CCI, and age, only the very high-risk group was significantly associated with 30-day mortality (OR 5.54 95%CI 1.73–17.79). Elevated risks for secondary outcomes were also observed solely in the very high-risk group, even after adjusting for age and sepsis (Supplementary Table S4).

### 3.3. Association between RFS Risk Factors and Outcomes

**Figure 3:**
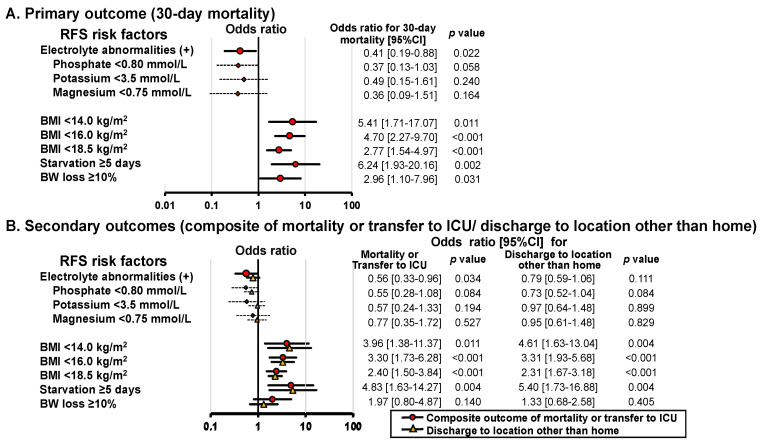
Univariable analysis of the relationship between RFS factors and the primary and secondary outcomes. The results are presented as odds ratios with 95% CIs and *p*-values. BMI, body mass index; BW, body weight; CI, confidence intervals; ICU, intensive care unit; RFS, refeeding syndrome.

**Figure 4:**
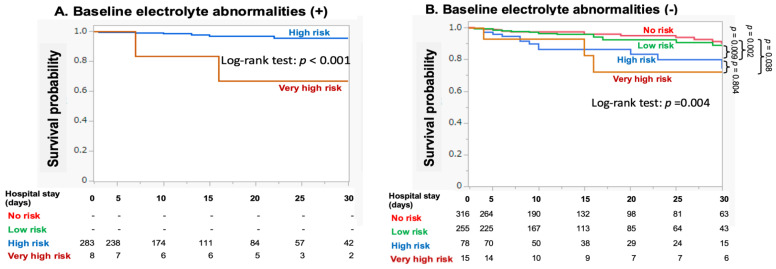
Kaplan-Meier survival analysis for 30-day mortality in patients with or without baseline electrolyte abnormalities.

The impact of each RFS risk factor on 30-day mortality after HDU admission was assessed using univariate logistic regression analysis. Low BMI, starvation period, and BW loss were associated with increased 30-day mortality (Figure 3A). In contrast, electrolyte abnormalities on admission, particularly hypophosphatemia, were inversely associated with 30-day mortality. Similar results were obtained for secondary outcomes (Figure 3B). In multivariate analysis, which included age, BMI, starvation period, BW loss, and sepsis as covariables, electrolyte abnormalities remained a negative factor for 30-day mortality (OR = 0.45 [95%CI: 0.20–0.98], *p* = 0.043). Furthermore, in patients without electrolyte abnormalities upon HDU admission, the survival probability for the high-risk group decreased over time, with significant reductions compared to the no- or low-risk groups (Figure 4). Neither alcohol abuse, insulin treatment, chemotherapy, antacids, nor diuretics were related to 30-day mortality (0% [*p* = 1.0], 6.1% [*p* = 0.774], 8.3% [*p* = 0.484], 5.5% [*p* = 0.926], 6.8% [*p* = 0.334], respectively).

## 4. Discussion

In the present study, we evaluated the distribution of patients who were urgently admitted to the HDU based on the modified NICE RFS risk criteria [6]. The results indicated that approximately 40% of patients were categorized into the high-risk or very high-risk groups (Table 2). The very high-risk group emerged as an independent prognostic predictor for all outcomes assessed in this study (i.e., 30-day mortality, composite outcome of in-hospital mortality or ICU transfer censored at 30-days, and discharge to locations other than home), whereas neither the low- nor the high-risk groups showed an elevated risk for these outcomes. Although electrolyte abnormalities are defined as a major RFS risk factor in the modified NICE criteria [6], our study suggests that they have possibly even favorable associations when assessed at HDU admission (Figure 3), contrasting with our findings in previous ICU patients [12].

Although the pathogenesis of RFS is not fully understood, there appears to be heterogeneity in the severity of patients’ vital status, which may influence the incidence of RFS [2]. Kraaijenbrink et al. found that 54% of patients acutely admitted to the department of internal medicine were at risk of developing RFS when evaluated using the original NICE criteria [15]. Using the modified NICE criteria, we previously demonstrated that 48.5% of critically ill patients requiring ICU admission were at high risk or very high risk of developing RFS (Supplementary Table S3) [12]. In the present study, 37.8% of HDU patients were classified as high-risk, and 2.4% as very high-risk. Although our current study, like our previous work, focused on the risks of developing RFS rather than its occurrence of RFS, it is reasonable to infer that a substantial number of patients in the HDU and the ICU are at risk for developing RFS, with a higher risk observed among ICU patients. This is consistent with a systematic review reporting the highest incidence of RFS among critically ill patients admitted to the ICU [2].

There is controversy regarding the impact of RFS on the prognosis of hospitalized patients. Some studies have reported that RFS is associated with worse prognosis in critically ill and in-hospital patients [7,8,9,28], while others have found no association between RFS and mortality [31,32]. These conflicting observations may be attributed to factors such as the diagnostic criteria for RFS, the situation, and the patients’ background. Importantly, management policies differ between critically ill patients and those who are less critically ill. The American Society for Parenteral and Enteral Nutrition provides recommendations and an evidence-based, consensus-supported algorithm targeting less critically ill patients [5,6]. These guidelines emphasize assessing risk before nutritional therapy, managing energy restriction, and evaluating electrolytes based on risk categories. In contrast, the European Society for Clinical Nutrition and Metabolism guidelines for critically ill patients recommend nutritional restriction and frequent electrolyte assessment following the detection of hypophosphatemia [13], with improved mortality reported in patients with hypophosphatemia in a randomized control trial [4]. Thus, the prognostic impact and implications of each risk group, risk factor, and electrolyte abnormality may vary depending on the severity of the illness.

Intriguingly, the present study found that neither the low-risk nor high-risk patient categories were associated with serious events, including primary and secondary outcomes (Figure 2, Table 2, and Supplementary Table S4). This result differs from our previous findings where risk was associated with outcomes in ICU patients [12] (Supplementary Table S3). In HDU patients, the modified NICE criteria may be more effective due to its separate category for very high risk, which conventional NICE criteria might not adequately identify [10]. Alternatively, since the modified NICE stratifies patients into four risk categories, the higher mortality observed among the very high-risk groups in both HDU and ICU settings suggests that more sophisticated nutritional care is needed to improve the prognosis of the group, particularly to differentiate HDU patients from other groups. To mitigate bias in the very high-risk group in this study, we excluded patients who did not wish to receive aggressive treatment. Patients in the end stage of their condition often have severe malnutrition, which may likely categorize them into the very high-risk group.

The present study demonstrated that the relation of electrolyte abnormalities to primary (i.e., 30-day mortality) or secondary outcomes were markedly different from those of other nutritional parameters (i.e., BMI, starvation period, and BW loss, Figure 3). Furthermore, the association between electrolyte abnormalities and 30-day mortality differed between HDU and ICU patients (Supplementary Figure S1). It is of note that survival probability in each risk group decreased in a risk-dependent manner when evaluated in patients with no electrolyte abnormalities at HDU admission (Figure 4B) or when electrolyte abnormalities were excluded from the modified NICE criteria (Supplementary Figure S2). This configuration markedly differs from that constructed using the original dataset (Figure 2). It appears that electrolyte abnormalities, when detected earlier at HDU admission play a paradoxically protective role in the development of serious outcomes, suggesting a need for distinct management approaches for RFS depending on the severity of the condition.

Although it remains unclear why electrolyte abnormalities have a different association with primary and secondary outcomes among HDU and ICU patients, several factors may be contributing to this issue. Although most studies have reported that hypophosphatemia is associated with poor outcomes [21,33,34], a recent systematic review revealed that, in sepsis, hyperphosphatemia was associated with increased mortality. In contrast, hypophosphatemia tended to reduce mortality [35]. The influence may differ across target populations. Notably, the cutoff values for phosphorus levels were different across studies [11,21]. In this study, a 0.32 mmol/L (1.0 mg/dL) increase in serum phosphorus levels was significantly associated with 30-day mortality in the univariate analysis (OR 1.44, 95% CI 1.19–1.72). Additionally, the ROC curve analysis identified a phosphorus cutoff value of 1.07 mmol/L for predicting 30-day mortality (AUC: 0.70) (Supplementary Figure S3). In this study, we evaluated only the presence of low electrolyte abnormalities according to the NICE criteria, which may have influenced positive outcomes. Previous research has reported that more severe hypophosphatemia (<0.32 mmol/L) is associated with higher mortality compared to less severe hypophosphatemia [36]. Furthermore, other studies have found that hyperphosphatemia with increasingly elevated levels is more strongly associated with mortality than hypophosphatemia [37]. Further research is needed to clarify the appropriate electrolyte cutoff values and the impact of electrolyte abnormalities across different diseases and settings. In patients with electrolyte abnormalities at HDU admission, close monitoring and early correction are typically anticipated [5,6,10], and in our HDU, these measures are carried out using protocol (Supplementary Table S2). Conversely, patients without apparent electrolyte abnormalities at HDU admission might develop RFS unnoticed due to less frequent blood sampling compared to the ICU setting [13,38]. More accurate assessment of RFS risks might be achieved with more frequent electrolyte evaluations after HDU admission, as hypophosphatemia often occurs within 12 h of admission [38]. Finally, factors other than electrolyte abnormalities, such as nutritional screening, assessment of BMI, and the duration of starvation [39,40,41], might play a more significant role in the development of RFS and subsequent serious outcomes.

## 5. Limitations

Since the present study was conducted at a single center, caution is needed when extrapolating our results to other studies with different populations. However, comparing patients urgently admitted to the HDU and ICU within the same hospital may provide higher comparability than comparisons across different facilities. Although 1741 patients admitted to the HDU were surveyed, 638 were excluded due to missing values, particularly for phosphate and magnesium. These excluded subjects may not have undergone a detailed assessment for RFS risk because medical professionals considered them to be at low risk of developing RFS. As a result, the proportion of the high-risk or very high-risk group may be overestimated and have different impacts on outcomes due to selection bias. Furthermore, most patients had received nutritional intervention and electrolyte correction based on our protocol (Supplementary Table S2) before RFS risks were assessed, which could reduce the number of patients with poor prognosis. Alternatively, patients with missing electrolyte data at HDU admission were not supposed to receive electrolyte supplementation until the next assessment, which allowed the assumption that this subgroup could be analyzed together with that with normal electrolyte values. Thus, we constructed an analysis model that covered both the included (*n* = 955) and excluded cases (*n* = 638) for evaluation. Both Kaplan–Meier and logistic regression analyses revealed nearly the same results as those demonstrated using the original subpopulations (Supplementary Figures S4 and S5). Another limitation of our study is that while this study evaluated outcomes over one month, the long-term prognosis of this disease remains undetermined. Finally, strict energy restriction (5–10 kcal/kg/day) had been recommended for high-risk and very high-risk groups during the study period [6,10]. A recent systematic review indicated that strict energy restriction may lead to more extended hospital stays in patients with malnutrition and RFS [42]. Furthermore, a large randomized controlled trial (RCT) involving non-ICU patients with nutritional risk demonstrated that aggressive energy and protein supplementation reduced mortality compared to standard care [43]. This suggests that the strict nutritional restriction imposed on the very high-risk group with malnutrition in our study may have contributed to worse outcomes.

In the future, there is a need to develop new RFS risk criteria that categorize patients based on severity. Additionally, further research is required to determine the optimal nutritional intake for patients at risk of RFS, particularly those with comorbid malnutrition.

## 6. Conclusions

Approximately 40% of patients who were less critically ill but admitted urgently to the HDU were classified into the high-risk or very high-risk categories for developing RFS. Only the very high-risk group was associated with serious events, including mortality and secondary outcomes. In contrast, the high-risk group was not linked to these serious outcomes, likely due to the association between electrolyte abnormalities at HDU admission and better outcomes. These observations suggest the need for distinct nutritional therapy strategies based on the severity of the patients’ conditions.

## Figures and Tables

**Table 1:** Characteristics of the patients in each risk group based on modified NICE criteria.

	Total	No Risk	Low Risk	High Risk	Very High Risk	*p* Value
Number of patients in each risks, *n* (%)	*n* = 955	*n* = 316 (33.1)	*n* = 255 (26.7)	*n* = 361 (37.8)	*n* = 23 (2.4)	
Age, median [IQR], year	75 [62–83]	70 [52–80]	76 [68–84]	77 [65–84]	75 [65–80]	<0.001
Male sex, *n* (%)	528 (55.3)	177 (56.0)	136 (53.3)	202 (56.0)	13 (56.5)	0.909
BMI, median [IQR], kg/m^2^	22.0 [19–24]	23.0 [21–25]	22.0 [19–24]	20.9 [18–24]	13.6 [13–14]	<0.001
CCI, median [IQR]	1 [0–1]	1 [0–2]	1 [1–2]	1 [1–2]	1 [1–2]	<0.001
Pre-existing illness						
Chronic kidney disease on dialysis, *n* (%)	44 (4.6)	8 (2.5)	20 (7.8)	16 (4.4)	0 (0.0)	0.016
Malignancy, *n* (%)	37 (3.9)	14 (4.4)	7 (2.8)	16 (4.4)	0 (0.0)	0.500
Psychiatric, *n* (%)	7 (8.1)	30 (9.5)	22 (8.6)	22 (6.1)	3 (13.0)	0.303
Anorexia nervosa, *n* (%)	0 (0.0)	0 (0.0)	0 (0.0)	0 (0.0)	0 (0.0)	
Living place before HDU admission						0.892
Home, *n* (%)	927 (97.1)	309 (97.8)	245 (96.1)	350 (97.0)	23 (100.0)	
Other hospital, *n* (%)	10 (1.0)	3 (1.0)	3 (1.2)	4 (1.1)	0 (0)	
Nursing facility, *n* (%)	17 (1.8)	4 (1.3)	7 (2.8)	6 (1.7)	0 (0)	
Ambulance use, *n* (%)	495 (52.8)	153 (48.4)	137 (53.7)	195 (54.0)	10 (43.5)	0.365
Emergency surgery, *n* (%)	71 (7.4)	35 (11.1)	16 (6.3)	19 (5.3)	1 (4.4)	0.024
Primary diagnosis						<0.001
Cardiovascular, *n* (%)	144 (15.1)	34 (10.8)	59 (23.1)	47 (13.0)	4 (17.4)	
Respiratory, *n* (%)	144 (15.1)	41 (13.0)	33 (12.9)	63 (17.5)	7 (30.4)	
Digestive, *n* (%)	67 (7.0)	21 (6.6)	15 (5.9)	29 (8.0)	2 (8.7)	
Neurology, *n* (%)	257 (26.9)	108 (34.2)	63 (24.7)	83 (23.0)	3 (13.0)	
Infection, *n* (%)	33 (3.5)	6 (1.9)	8 (3.1)	18 (5.0)	1 (4.3)	
Trauma, *n* (%)	90 (9.4)	46 (14.6)	17 (6.7)	26 (7.2)	1 (4.3)	
Metabolic, *n* (%)	100 (10.5)	28 (8.9)	26 (102)	43 (11.9)	3 (13.0)	
Hematologic, *n* (%)	10 (1.1)	0 (0.0)	6 (2.4)	3 (0.8)	1 (4.3)	
Genitourinary, *n* (%)	28 (2.9)	8 (28.6)	4 (1.6)	16 (4.4)	0 (0.0)	
Skin musculoskeletal, *n* (%)	82 (8.6)	24 (29.3)	24 (9.4)	33 (9.1)	1 (4.3)	
Diagnosis of sepsis, *n* (%)	99 (10.4)	22 (7.0)	26 (10.2)	48 (13.3)	3 (13.0)	0.059
Treatment on admission						
Mechanical ventilation with intubation and circulatory support	0 (0.0)	0 (0.0)	0 (0.0)	0 (0.0)	0 (0.0)	

BMI, body mass index; CCI, Charleson Comorbidity Index, HDU, high dependency unit; IQR, interquartile range; NICE, National Institute for Health and Clinical Excellence.

**Table 2:** NICE risk factors and risk categorization.

		Modified NICE RFS Risk Group				
	Total	No Risk	Low Risk	High Risk	Very High Risk	*p*-Value
*n* (%)	955	316 (33.1)	255 (26.7)	361 (37.8)	23 (2.4)	
BMI (kg/m^2^)						<0.001
16 kg/m^2^ ≤ BMI < 18.5 kg/m^2^	128 (13.4)	0	49 (5.1)	79 (8.3)	0	
14 kg/m^2^ ≤ BMI < 16 kg/m^2^	43 (4.5)	0	0	40 (4.2)	3 (0.3)	
BMI < 14 kg/m^2^	18 (1.9)	0	0	0	18 (1.9)	
Weight loss during the 6 months						<0.001
>10% and ≤15%	12 (1.3)	0	3 (0.3)	6 (0.6)	3 (13.0)	
>15% and ≤20%	5 (0.5)	0	0	4 (0.4)	1 (0.1)	
>20%	5 (0.5)	0	0	0	5 (0.5)	
Little or no nutritional intake (duration)						<0.001
>5 and ≤10 days	9 (0.9)	0	2 (0.2)	6 (0.6)	1 (0.1)	
>10 and ≤15 days	3 (0.3)	0	0	3 (0.3)	0	
>15 days	3 (0.3)	0	0	0	3 (0.3)	
Low baseline levels of electrolytes on admission	291 (30.5)	0	0	283 (29.6)	8 (0.8)	<0.001
Phosphate < 0.80 mmol/L	174 (18.2)	0	0	173 (18.1)	1 (0.1)	<0.001
Potassium < 3.5 mmol/L	105 (11.0)	0	0	99 (10.4)	6 (0.6)	<0.001
Magnesium < 0.75 mmol/L	95 (9.9)	0	0	91 (9.5)	4 (0.4)	<0.001
Alcohol abuse ^†^	15 (1.6)	0	8 (0.8)	5 (0.5)	2 (0.2)	<0.001
Insulin treatment	66 (6.9)	0	40 (4.2)	25 (2.6)	1 (0.1)	<0.001
Chemotherapy	12 (1.3)	0	6 (0.6)	6 (0.6)	0	0.064
Antacids	201 (21.0)	0	99 (10.4)	89 (9.3)	13 (1.4)	<0.001
Diuretics	176 (18.4)	0	110 (11.5)	65 (6.8)	1 (0.1)	<0.001
30-day mortality, *n* [% of each risk group]	51 [5.3%]	14 [4.4%]	14 [5.5%]	18 [5.0%]	5 [21.7%]	0.005
Odds ratio [95%CI]						
Univariate		1.0 (Ref.)	1.25 [0.58–2.67]	1.13 [0.55–2.31]	5.99 [1.94–18.48]	
Multivariate, adjusted with sepsis		1.0 (Ref.)	1.17 [0.54–2.51]	0.98 [0.47–2.03]	5.63 [1.77–17.84]	
Multivariate, adjusted with sepsis and CCI		1.0 (Ref.)	0.91 [0.41–2.02]	0.91 [0.43–1.89]	5.53 [1.73–17.66]	
Multivariate, adjusted with sepsis, CCI, and age (Primary analysis)		1.0 (Ref.)	0.86 [0.38–1.88]	0.81 [0.38–1.68]	5.54 [1.73–17.79]	

BMI, body mass index; CCI, Charleson Comorbidity Index; CI, confidence interval; NICE, National Institute for Health and Clinical Excellence. ^†^ Alcohol abuse was defined as daily alcohol consumption of more than four standard drinks for men or more than three standard drinks for women.

## Data Availability

The datasets generated and analyzed in this study are available from the corresponding author upon reasonable request due to ethical reasons. Approval from the ethics committee is required before providing access to the data.

## Supplementary Materials

The following supporting information can be downloaded at: https://www.mdpi.com/article/10.3390/nu16193287/s1, Table S1. NICE risk factors and modified NICE criteria. Table S2. Correction and re-evaluation protocol for electrolyte abnormality in the HDU. Table S3. Comparison of the distribution and 30-day mortality across each risk group in HDU and ICU. Table S4. Effects of RFS risk categories on secondary outcomes. Figure S1. Comparison of the impact of various RFS risk factors on 30-day mortality between HDU and ICU patients. Figure S2. Survival analyses for 30-day mortality using revised criteria (with the item on electrolyte abnormalities eliminated from the modified NICE criteria). Figure S3. Receiver operating characteristic curve for serum phosphate levels predicting 30-day mortality. Figure S4. Survival probabilities in patients with or without electrolyte abnormalities. Figure S5. Survival probabilities in patients with or without hypophosphatemia/hypomagnesemia/hypokalemia.
