# Supplementary material for: Association between Poor Outcomes and Risk of Refeeding Syndrome among Patients Urgently Admitted to the High Dependency Unit: A Single-Center Cohort Study in Japan
Source: Nutrients. 2024 Sep 28;16(19):3287. doi: 10.3390/nu16193287 (PMC11478408; doi:10.3390/nu16193287)

Supplementary Table S1. NICE risk factors and modified NICE criteria.

**Major risk factors**	
$14\ kg/m^2 \leq$ BMI $<16\ kg/m^2$	
Weight loss during 6 months >15% and ≤20%	
Little or no nutritional intake for >10 days and ≤15 days	
Low baseline levels of electrolytes on admission: phosphate <0.80 mmol/L, potassium <3.5 mmol/L, or magnesium <0.75 mmol/L	
**Minor risk factors**	
$16\ kg/m^2 \leq$ BMI $<18.5\ kg/m^2$	
Weight loss during 6 months of >10% and ≤15%	
Little or no nutritional intake for >5 and ≤10 days	
Alcohol abuse† or receive insulin, chemotherapy, antacids, or diuretics before admission.	
**Modified NICE criteria**	
**No risk**	No minor or major risk factors
**Low risk**	One minor risk factor
**High risk**	One major risk factors or two minor risk factors
**Very high risk**	Meeting any of the following factors:
	BMI $<14\ kg/m^2$
	Weight loss during 6 months >20%
	Little or no nutritional intake for >15 days

NICE, National Institute for Health and Clinical Excellence; BMI, body mass index.

†Alcohol use disorder was defined as daily alcohol consumption of more than four standard drinks for men or more than three standard drinks for women.

Supplementary Table S2. Correction and re-evaluation protocol for electrolyte abnormality in the HDU.

**Protocol for electrolyte abnormality in the HDU**				
Electrolyte Abnormality	Serum Level	Intervention	Dosage	Route of Administration
Hypophosphatemia	< 0.80 mmol/L	Sodium Phosphate	10 mmol	Intravenous (IV)
Hypokalemia	< 3.5 mmol/L	Potassium Chloride	20 mEq (Oral if no central venous line)	Intravenous (IV) or Oral
Hypomagnesemia	< 0.75 mmol/L	Magnesium Sulfate	20 mEq	Intravenous (IV)
**Re-evaluation strategy for electrolyte abnormality**				
Serum levels should be rechecked every 8-12 hours after the administration of electrolytes to ensure proper correction. If needed, corrections should be repeated.				

Supplementary Table S3. Comparison of the distribution and 30-day mortality across each risk group in HDU and ICU.

	Setting	Total	No risk	Low risk	High risk	Very high risk
**Distribution of each risk group, n, (%)**	**HDU**	955	361 (33.1%)	255 (26.7%)	361 (37.8%)	23 (2.4%)
	**ICU**	542	140 (25.8%)	139 (25.7%)	252 (46.5%)	11 (2.0%)
**30-day mortality of each risk group, n, (%)**	**HDU**	955	14 (4.4%)	14 (5.5%)	18 (5.0%)	5 (21.7%)
	**ICU**	542	7 (5.0%)	10 (7.2%)	41 (16.3%)	3 (27.3%)

HDU, high dependency unit; ICU, intensive care unit.

Supplementary Table S4. Effects of RFS risk categories on secondary outcomes.

n, (%)	RFS risk category<br>No risk<br>n=316 (33.1)	Low risk<br>n=255 (26.7)	High risk<br>n=361 (37.8)	Very high risk<br>n=23 (2.4)	*p* value
**Composite outcome of 30-day mortality or transfer to ICU, n (% of each risk category)**	19 (6.0%)	27 (10.6%)	27 (7.5%)	7 (30.4%)	<0.001
Odds ratio [95%CI]					
Univariate	1.0 (Ref.)	1.85 [1.00-3.41]	1.26 [0.68-2.31]	6.84 [2.51-18.62]	
Multivariate, adjusted with sepsis, CCI, and age	1.0 (Ref.)	1.46 [0.77-2.73]	1.01 [0.54-1.88]	6.08 [2.19-16.84]	
**Discharge to location other than home, n (% of each risk category)**	105 (33.2%)	93 (36.5%)	138 (38.2%)	15 (65.2%)	0.018
Odds ratio [95%CI]					
Univariate	1.0 (Ref.)	1.15 [0.81-1.63]	1.24 [0.90-1.70]	3.77 [1.54-9.16]	
Multivariate, adjusted with sepsis, CCI, and age	1.0 (Ref.)	0.87 [0.60-1.24]	1.00 [0.71-1.39]	3.20 [1.29-7.91]	

CI, confidence intervals; CCI, Charleson Comorbidity Index; ICU, intensive care unit; RFS, refeeding syndrome.

Supplementary Figure S1. Comparison of the impact of various RFS risk factors on 30-day mortality between HDU and ICU patients.

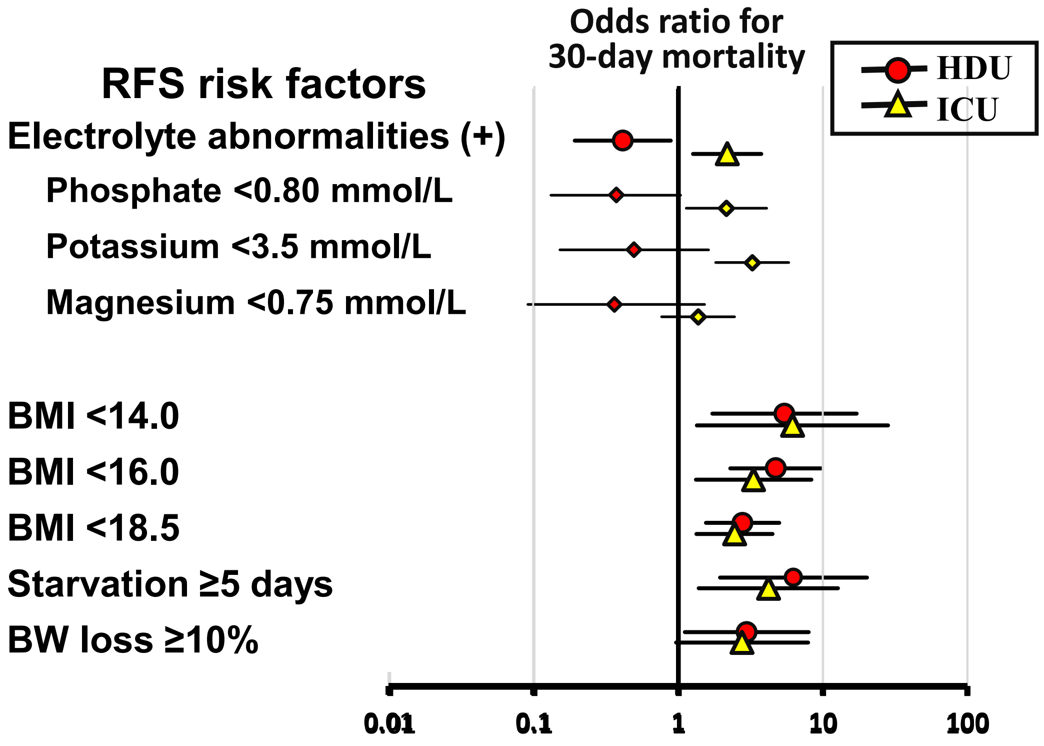

Odds ratio [95%CI] for 30-day mortality

HDU	*p*-value	ICU*	*p*-value
0.41 [0.19-0.88]	0.022	2.19 [1.27-3.76]	0.005
0.37 [0.13-1.03]	0.058	2.16 [1.14-4.10]	0.018
0.49 [0.15-1.61]	0.240	3.26 [1.82-5.82]	<0.001
0.36 [0.09-1.51]	0.164	1.38 [0.77-2.46]	0.281
5.41 [1.71-17.07]	0.011	6.17 [1.35-28.25]	0.019
4.70 [2.27-9.70]	<0.001	3.33 [1.33-8.35]	0.010
2.77 [1.54-4.97]	<0.001	2.46 [1.34-4.50]	0.004
6.24 [1.93-20.16]	0.002	4.21 [1.39-12.74]	0.011
2.96 [1.10-7.96]	0.031	2.77 [0.97-7.92]	0.057

Marked differences in odds ratio for electrolyte abnormalities, particularly phosphate, were observed between HDU and ICU patients whereas there was no difference in nutritional parameters or BMI between these patient populations.

*, The results pertaining to ICU were obtained from the de novo analysis of the data in our previous study (Yoshida M, et al., Clin Nutrition 2021; 40: 1207-1213, Table 4).

BMI, body mass index; BW, body weight; CI, confidence intervals; HDU, high dependency unit; ICU, intensive care unit; RFS, refeeding syndrome.

Supplementary Figure S2. Survival analyses for 30-day mortality using revised criteria (with the item on electrolyte abnormalities eliminated from the modified NICE criteria).

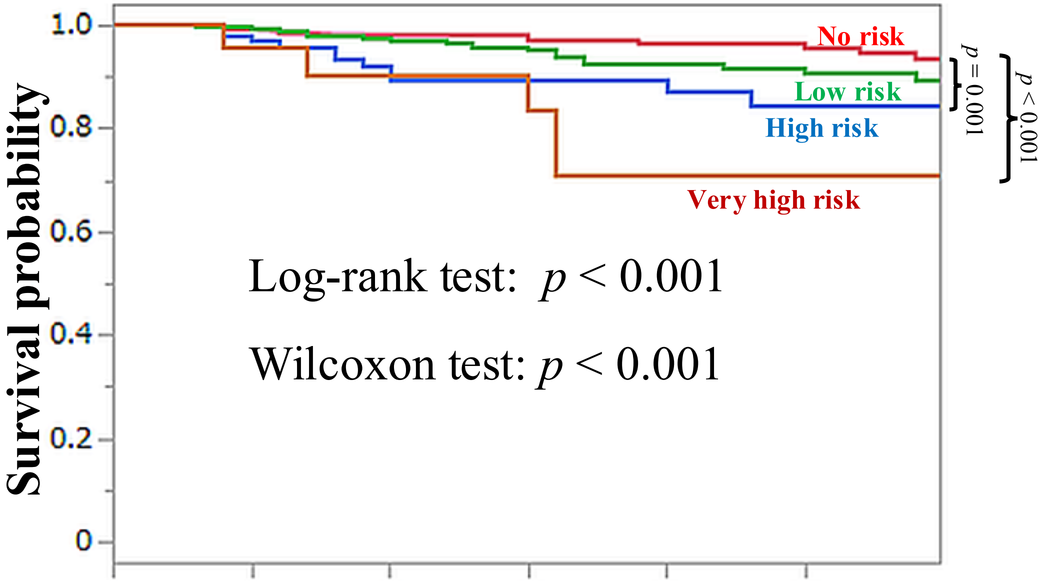

Hospital stay (days)	0	5	10	15	20	25	30
No risk	471	392	275	182	135	107	84
Low risk	359	315	240	160	120	85	59
High risk	102	90	66	52	41	34	20
Very high risk	23	20	15	14	11	9	9

We adopted a revised criteria for RFS in which the item on electrolytes was eliminated from the modified NICE criteria. Kaplan-Meier analysis showed a risk-dependent decrease in survival probability in patients admitted to the HDU (see also Figure2). NICE, National Institute for Health and Clinical Excellence; HDU, high dependency unit.

Supplementary Figure S3. Receiver operating characteristic curve for serum phosphate levels predicting 30-day mortality.

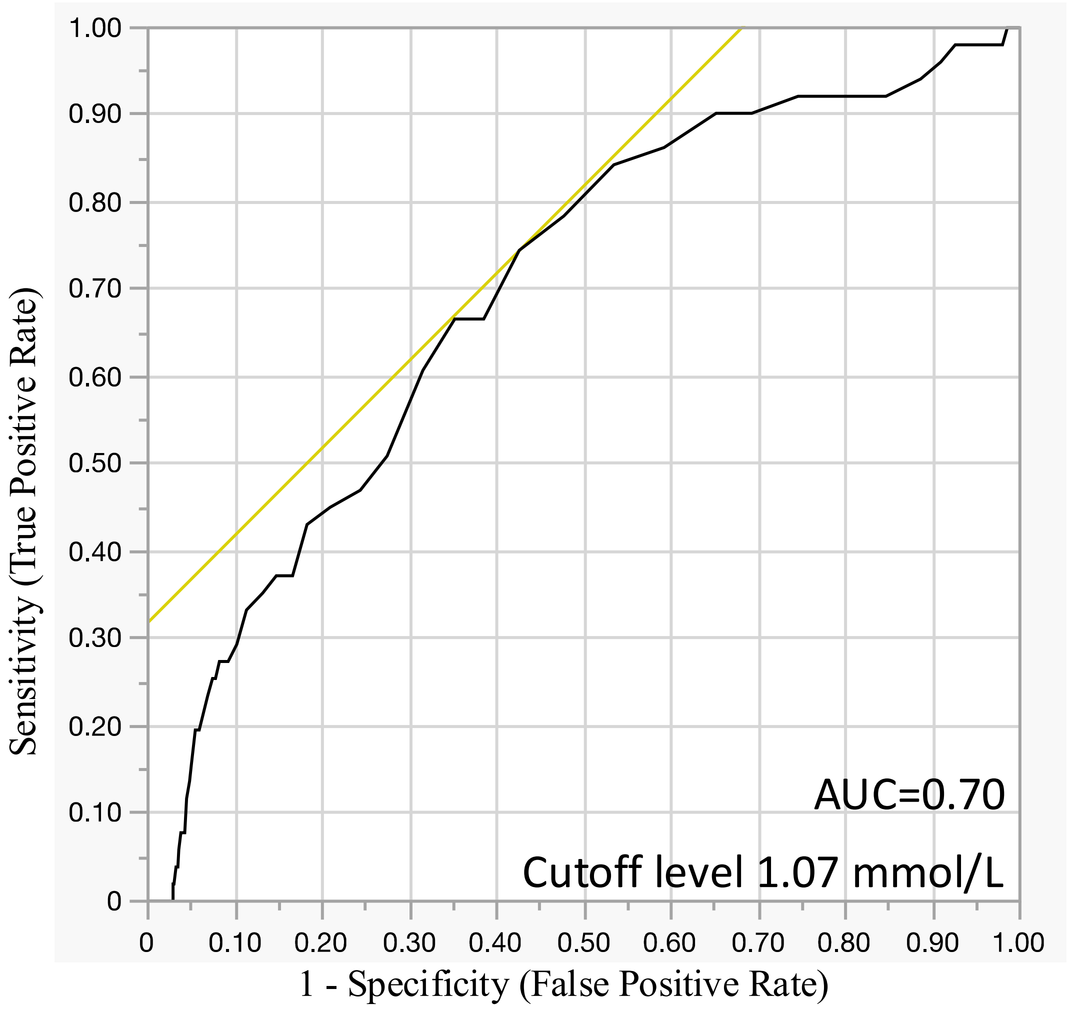

We analyzed the Receiver Operating Characteristic curve for serum phosphate levels predicting 30-day mortality. The Area Under the Curve (AUC) and the serum phosphate cutoff value are indicated.

Supplementary Figure S4. Survival probabilities in patients with or without electrolyte abnormalities.

A. Subgroup with electrolyte abnormalities, n=423

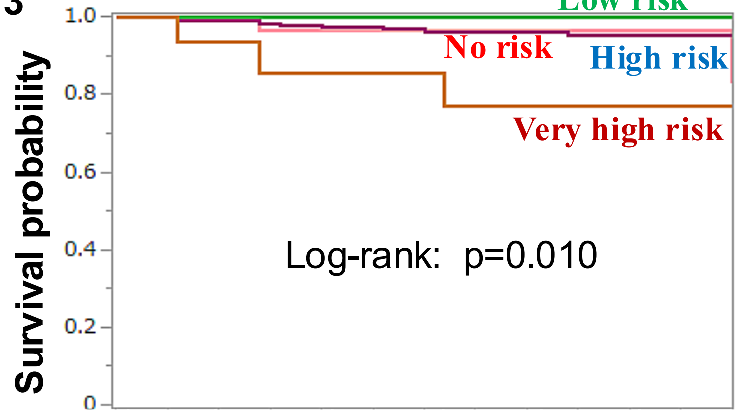

B. Subgroups with no electrolyte abnormalities + missing electrolyte measurement, n=1,318

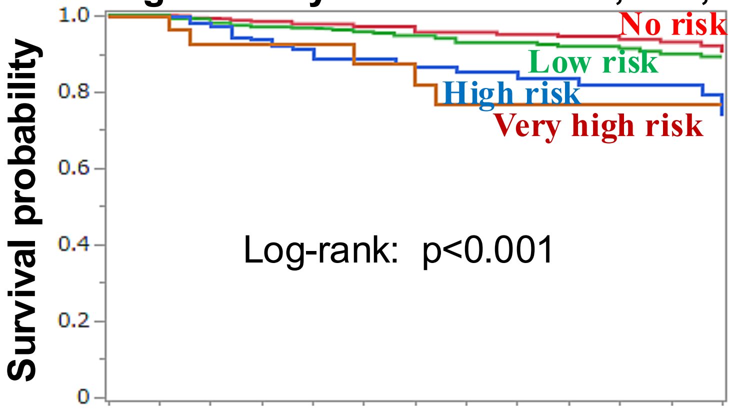

Hospital stay (days)	A: 0	5	10	15	20	25	30	B: 0	5	10	15	20	25	30
No risk	35	32	24	19	13	10	7	591	511	366	254	175	135	99
Low risk	34	33	28	22	14	11	8	542	492	392	277	204	148	106
High risk	337	283	214	138	107	74	56	154	139	103	99	56	42	29
Very high risk	15	13	12	11	9	6	2	28	26	21	16	13	13	10

C. Odds ratio for 30-day mortality

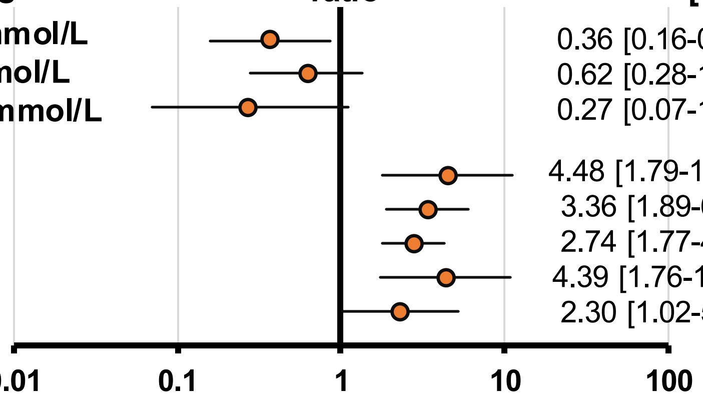

RFS risk factors	Odds ratio [95%CI]	p value
Phosphate <0.80 mmol/L	0.36 [0.16-0.85]	0.019
Potassium <3.5 mmol/L	0.62 [0.28-1.36]	0.231
Magnesium <0.75 mmol/L	0.27 [0.07-1.12]	0.071
BMI <14.0 kg/m^2	4.48 [1.79-11.21]	0.001
BMI <16.0 kg/m^2	3.36 [1.89-6.00]	<0.001
BMI <18.5 kg/m^2	2.74 [1.77-4.26]	<0.001
Starvation ≥5 days	4.39 [1.76-11.00]	0.002
BW loss ≥10%	2.30 [1.02-5.19]	0.045

Both included and excluded subgroups were combined and 30-day survival probabilities for various RFS risk were evaluated in patients with or without electrolyte abnormalities + missing electrolyte data.

Supplementary Figure S5. Survival probabilities in patients with or without hypophosphatemia/ hypomagnesemia/ hypokalemia.

A-1. Subgroup with hypophosphatemia, n=367

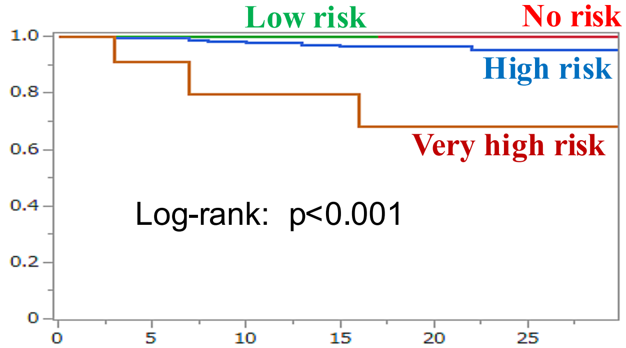

A-2. Subgroup with no hypophosphatemia + missing phosphate measurement, n=1,374

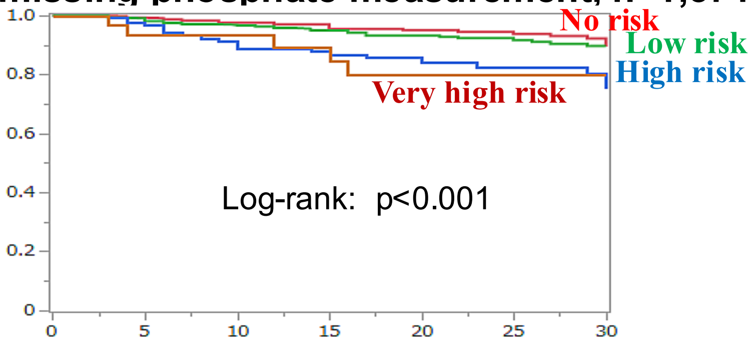

B-1. Subgroup with hypomagnesemia, n=110

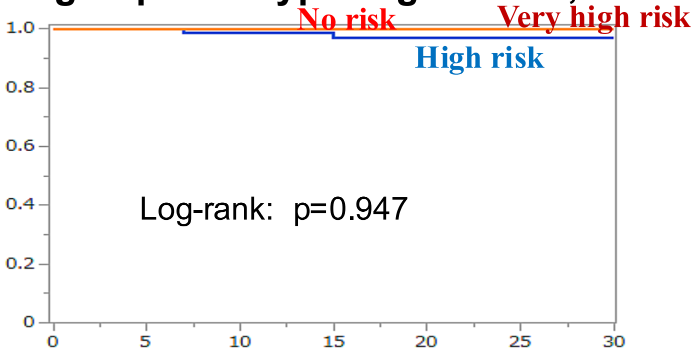

B-2. Subgroup with no hypomagnesemia + missing magnesium measurement, n=1,401

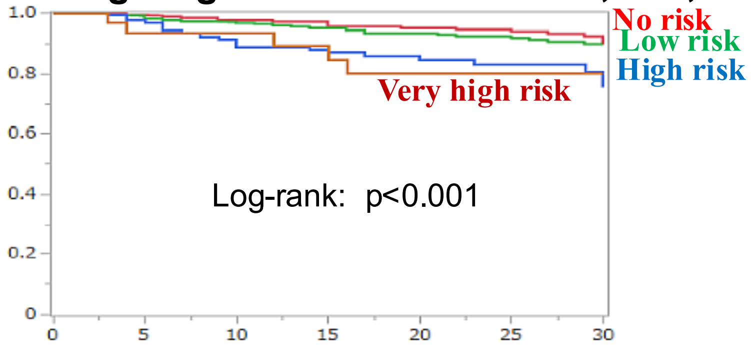

C-1. Subgroup with hypokalemia, n=192

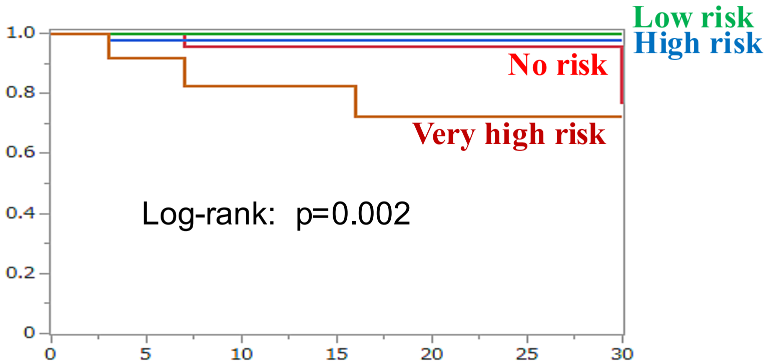

C-2. Subgroup with no hypokalemia + missing potassium measurement, n=1,340

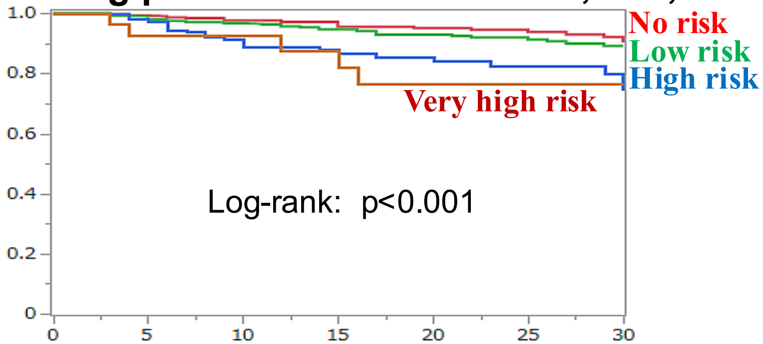

Supplement: Supplementary file 1 [file nutrients-16-03287-s001.zip › nutrients-3198527-supplementary.pdf]
